# Disorders of nomenclature

**DOI:** 10.1111/cns.14541

**Published:** 2024-01-12

**Authors:** John C. O'Donnell

**Affiliations:** ^1^ Center for Neurotrauma, Neurodegeneration, and Restoration Corporal Michael J. Crescenz Veterans Affairs Medical Center Philadelphia Pennsylvania USA; ^2^ Center for Brain Injury and Repair, Department of Neurosurgery, Perelman School of Medicine University of Pennsylvania Philadelphia Pennsylvania USA

The cogent and appropriate application of established terms, definitions, and nomenclature are critical to the advancement of science. In contrast, the misuse of terms in service of inflating the clinical relevance of a preclinical study pollutes the scientific record and impairs translational efforts for as long as the misinformation persists. A recent paper in *CNS Neuroscience & Therapeutics* by Zhao et al. entitled “Transplantation of glutamatergic neuronal precursor cells in the paraventricular thalamus and claustrum facilitates awakening with recovery of consciousness” contains concerning misinformation and dubious interpretation of the reported findings.^1^ It centers around the claim that an injury‐associated 3‐min delay to the return of the vestibular righting reflex after anesthesia constitutes a preclinical model of traumatic Disorders of Consciousness (DoC) in mice. This claim is objectively false. Equating balance‐associated loss of righting reflex (LRR) with DoC demonstrates a fundamental misunderstanding of both phenomena. This false claim is the primary focus of the paper, and it is unfortunately not the only flaw. After discussing the matter with colleagues, I strongly recommend retraction of this paper to avoid further harm to the scientific record and to stem the spread of misinformation and confusion.

I study preclinical traumatic brain injury (TBI) and coma,^2^, ^3^ so I was initially excited to come across this paper. That excitement turned into shock when I discovered that the paper was built around the unsupportable claim that a brief delay to the return of the vestibular righting reflex could serve as a model of DoC. Since the mid‐1900s we have known that rotational acceleration of the head acting upon a brain of sufficient mass is necessary to induce traumatic loss of consciousness (brain mass × acceleration = diffuse injurious force). Loss of consciousness does not result from lateral acceleration, and protracted unconsciousness does not result from impact alone absent head rotational acceletaration.^4^, ^5^, ^6^, ^7^ Furthermore, anything less than 60 min of unconsciousness is mild TBI (not associated with DoC), and a few minutes of vestibular disorientation after impact/deep anesthesia (as reported in this study) is of little relevance. The paper also claims that the areas damaged by the cortical impact injury must be important for human DoC. To be clear, this injury applies force in a way that does not occur in human injury and therefore it is not indicative of the areas affected in human injuries.

Rodent LRR has been routinely quantified in many hundreds of reports of CCI and fluid percussion injury over many decades of research. However, unlike its portrayal in this paper, it has not been—and is not—associated with DoC. The delayed return of the rodent righting reflex is a vestibular effect commonly used after procedures requiring anesthesia to guide temperature support or analgesia administration. Aside from having no connection to DoC, its use as an outcome measure is controversial, and though some studies have included it in their results, high variability observed with LRR has been widely reported. As noted in a recent review comparing the prognostic value of brief loss of consciousness that may occur in human mild TBI versus LRR in rodent studies: “LRR is a highly heterogeneous measure subject to large individual variability, such that two rodents experiencing the same injury with identical parameters may have drastically different LRR.”^8^ Independent of the mischaracterization of brief LRR as a model of DoC, the extremely low variability in LRR reported in this paper is another anomaly that calls into question its integrity and reliability (Box 1).

BOX 1 **Examples of concerning reporting.**

Zhao et al. performed Controlled Cortical Impact (CCI) at a variety of impact depths to determine the appropriate settings for this study. Similar to many studies before them, a 4 mm deep cortical impact was reported to produce up to a 10 min delay of the vestibular righting reflex but was also largely fatal. Impact to 3.5 mm also produced quite a bit of mortality, so they settled on a 3 mm impact depth that resulted in an average delay of approximately 200 s for the return of the vestibular righting reflex, and then inexplicably called it a model of DoC. It should also be noted that these righting reflex delays (difference between injury and sham) had to be interpreted from the small *y*‐axis of Figure 1G because they do not report means or standard error for the duration of the delayed righting reflex. Indeed, the duration of the LRR on which they base their entire “model of DoC” is never reported in the text. This is not the only vague reporting present in the paper, as means and standard error do not appear anywhere in the text. Additional reasons for concern include errors with key results, such as reporting in the text that “the PVT‐IA group showed a statistically significant difference in the duration of LOC and the latency to wake (PVT‐IA group vs. NS group, *p* < 0.05; Figure 3D),” while Figure 3D clearly shows that these groups are not different. Overall, a close examination of the paper—beyond the disqualifying DoC claims—lead one to conclude that the overall accuracy, rigor, and integrity of this report are lacking.

I could not find anything in the literature to support the radical interpretation of LRR in rodents representing human coma or DoC in any capacity. There is nothing unique about the CCI, lesion injuries, or righting reflex testing applied in this study. In fact, much of the data presented seem to rehash the characterization of the CCI injury model that has been common knowledge for decades,^9^ inaccurately framed as a new characterization of a model of DoC. A scale of commonly observed recovery behaviors (Zhao et al., figure 1C) is included, which appears to be positioned as the unique feature that makes this a model of DoC, but it does not offer any additional value and this study is not the first to check for responsiveness in addition to the return of the righting reflex. There is no new or overlooked information that led to this sudden change in the interpretation of the LRR compared to decades of previous CCI studies. The only difference between this paper and the legions of previous studies utilizing the same techniques is the glaring misinterpretation of results in the context of DoC and the vague, sometimes inaccurate reporting.

There may have been valid data gathered during this study that could make up an accurate report on glutamatergic stem cells. Unfortunately, the inflation of clinical relevance via false claims of DoC modeling is infused throughout the entire paper, necessitating that the paper would need to be remade in its entirety. Correction via errata would need to be so extensive that retraction would be a mercy to all involved. This paper and others like it create confusion among researchers that causes real damage to a patient population in desperate need of new therapeutics. To end their abstract with the sentence: “Thus, these findings have the potential to provide a favorable basis for promoting awakening and recovery in patients with DOC,” and to end their paper with the sentence: “We thus offer a promising stem cell treatment for DOC that has great clinical research value,” is simply unethical. As a scientist, I am troubled by the disregard for accurate reporting and clinical relevance, but as someone who lost a close family member to complications associated with DoC after severe TBI, I am sincerely appalled. I appeal to the editors to retract this paper as soon as possible (Box 2).

BOX 2 **An appeal to the editors of CNS Neuroscience and Therapeutics.**

I applaud the editor's commitment thus far to preventing the spread of misinformation and protecting the scientific record in our email correspondence, but until there is action those expressions of integrity remain mere words. You graciously gave the authors the opportunity to make corrections via errata and they declined. At this point, retraction is the only remaining option. Allowing counterfactual reports to remain part of the scientific record is unacceptable, even if they are associated with a critique that points out their fallacies. To this point, decades ago a group attempted to injure rat brains via acceleration, apparently not aware of the fact that rodent brains are far below the necessary mass to make acceleration‐induced force damage achievable (mass × acceleration = force). Despite an associated letter to the editor that conclusively established that their approach conflicted with well‐established laws of physics, the paper was not retracted, and that misinformation continues to spread via the fatally flawed rodent CHIMERA (closed‐head impact model of engineered rotational acceleration) model that plagues our field to this day.^10^ The field of neurotrauma cannot afford another CHIMERA. I strongly encourage you to retract immediately, as this misinformation will continue to sew confusion and impair progress for as long as it spreads.

## CONFLICT OF INTEREST STATEMENT

The author declares no conflicts of interest.

## Data Availability

Data sharing is not applicable to this article as no new data were created or analyzed in this study.

## References

[1] Zhao T , Wei N , Li T , et al. Transplantation of glutamatergic neuronal precursor cells in the paraventricular thalamus and claustrum facilitates awakening with recovery of consciousness. CNS Neurosci Ther. 2023;29(7):1785‐1804. doi:10.1111/cns.14137 36880283 PMC10324366

[2] O'Donnell JC , Browne KD , Kilbaugh TJ , Chen HI , Whyte J , Cullen DK . Challenges and demand for modeling disorders of consciousness following traumatic brain injury. Neurosci Biobehav Rev. 2019;98:336‐346. doi:10.1016/j.neubiorev.2018.12.015 30550859 PMC7847278

[3] O'Donnell JC , Browne KD , Kvint S , et al. Multimodal neuromonitoring and neurocritical care in swine to enhance translational relevance in brain trauma research. Biomedicine. 2023;11(5):1336. doi:10.3390/biomedicines11051336 PMC10216032 37239007

[4] Denny‐Brown DE , Russell WR . Experimental concussion: (section of neurology). Proc R Soc Med. 1941;34(11):691‐692. 19992388 10.1177/003591574103401102 PMC1998140

[5] Langlois JA , Rutland‐Brown W , Wald MM . The epidemiology and impact of traumatic brain injury: a brief overview. J Head Trauma Rehabil. 2006;21(5):375‐378. 16983222 10.1097/00001199-200609000-00001

[6] Ommaya AK , Gennarelli TA . Cerebral concussion and traumatic unconsciousness. Correlation of experimental and clinical observations of blunt head injuries. Brain J Neurol. 1974;97(4):633‐654. 10.1093/brain/97.1.633 4215541

[7] Cullen DK , Harris JP , Browne KD , et al. A porcine model of traumatic brain injury via head rotational acceleration. Methods Mol Biol Clifton NJ. 2016;1462:289‐324. doi:10.1007/978-1-4939-3816-2_17 PMC5553045 27604725

[8] Berman R , Spencer H , Boese M , Kim S , Radford K , Choi K . Loss of consciousness and righting reflex following traumatic brain injury: predictors of post‐injury symptom development (a narrative review). Brain Sci. 2023;13(5):750. doi:10.3390/brainsci13050750 37239222 PMC10216326

[9] Dixon CE , Clifton GL , Lighthall JW , Yaghmai AA , Hayes RL . A controlled cortical impact model of traumatic brain injury in the rat. J Neurosci Methods. 1991;39(3):253‐262. doi:10.1016/0165-0270(91)90104-8 1787745

[10] Meaney DF , Margulies SS , Smith DH . Diffuse axonal injury. J Neurosurg. 2001;95(6):1108‐1110. doi:10.3171/jns.2001.95.6.1108 11765833

